# Montreal Veterinary Medical Association

**Published:** 1885-01

**Authors:** E. W. Hoare

**Affiliations:** Member of Reporting Committee


### MONTREAL VETERINARY MEDICAL ASSOCIATION.

The regular fortnightly meeting of the Montreal Veterinary Medical Association was held in the lecture room of the college on the 27th of November, Mr. M. C. Baker, V.S., President, in the chair. There were also present several visitors.

Mr. Wilcocks, of Guelph, Ont., was unanimously elected a member.

Mr. Mayor then communicated a case of Flatulent Colic which came under his notice last summer, and which was treated successfully by the operation of Enterotomy.

A discussion followed as to the advantages to be gained by following this plan of treatment, and also the danger run, but it was agreed that in bad cases it was the most successful treatment.

Mr. E. Wallis Hoare then read his paper on "Observations on the Examination of Horses as to Soundness." He described the difference between legal and practical soundness, and how, in the present day, the question of practical soundness was very important. He next described the method of examining for soundness, taking that adopted by the late Professor Dick. He also noticed the various defects likely to be met with, dwelling especially on the subject of spavin and the so-called coarse hocks, showing that a great deal of dispute frequently arose as to the question of the soundness of hocks.

The importance of testing for lameness and for the wind was dwelt on, and the advisability of removing the shoes in examining the feet for corns. The requirements of the examiner were also noticed, which were stated to be a thorough knowledge of the conformation of the horse, and quickness in observation and perception, so as at once to detect abnormalities. The importance of practice in this part of the profession was urged, as it was impossible to be proficient in it by mere learning out of books.

The importance of the veterinary surgeon being able to ride was dwelt on, especially if he practices in a district where many hunters or saddle horses are bought and sold, because many defects in action, etc., are noticed when the animal is ridden by the examiner himself.

A discussion followed as to the necessity of making such a thorough examination in all cases, but the essayist contended that without such an examination it was impossible to give a proper opinion on the horse.

The question of spavin and coarse hocks was also discussed, and the reasons why spavins situated far back on the hock were not so liable to cause lameness as those situated in front and outside.

After a few remarks by the President, the meeting then adjourned.

At the next meeting Mr. Clement, V.S., will communicate a case of Excision of a Cystic Tumor from the neck of a horse, and Mr. Lambe will read a paper on "Horse-shoeing."

E. W. Hoare,
Dec. 3d, 1884. Member of Reporting Committee.

